# Supplementary material for: Interstitial Lung Diseases in Israel: Large Variability in Close Geographic Proximity
Source: Diagnostics (Basel). 2025 Nov 2;15(21):2780. doi: 10.3390/diagnostics15212780 (PMC12608678; doi:10.3390/diagnostics15212780)
Supplement: Supplementary file 1 [file diagnostics-15-02780-s001.zip › diagnostics-3861268-supplementary.pdf]

Supplementary Materials

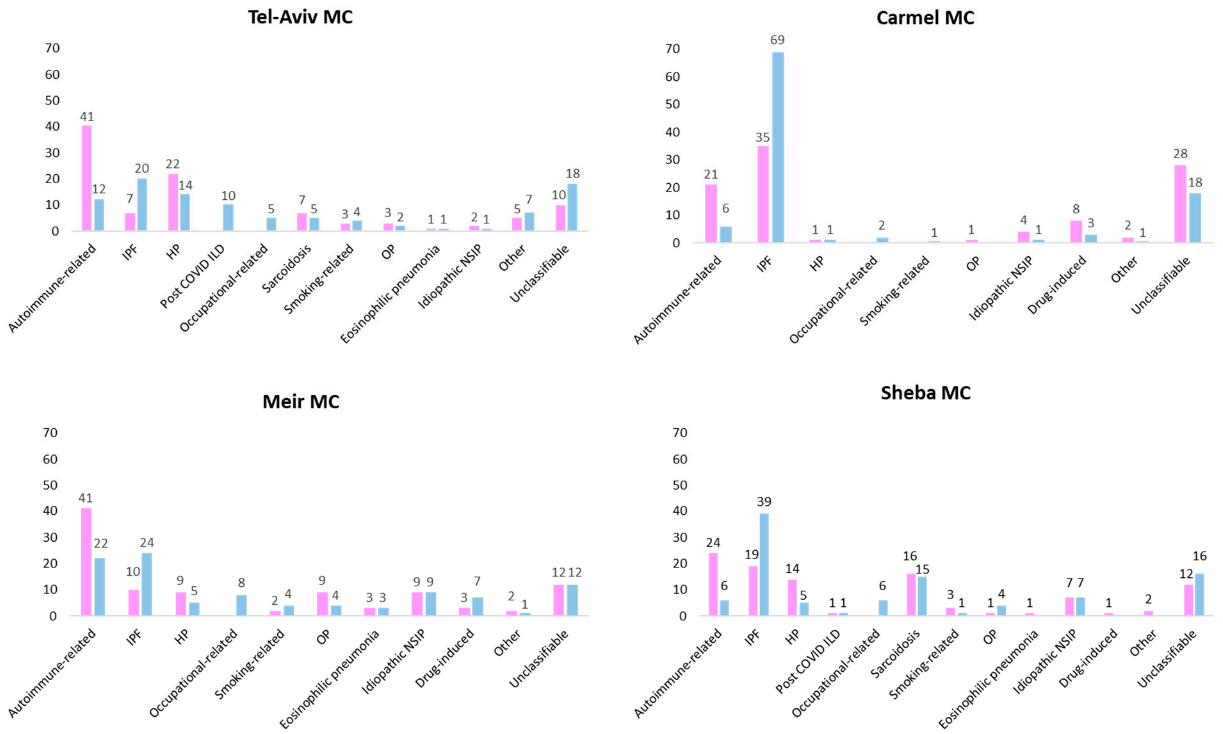

Figure S1. Interstitial lung disease diagnosis distribution according to sex in each medical center. Data are presented as percentage of subjects belonging to each of the ILD categories. Pink columns represent females while blue columns represent males.

Abbreviations: COVID coronavirus disease 2019, HP hypersensitivity pneumonitis, ILD interstitial lung diseases, IPF idiopathic pulmonary fibrosis, MC medical center, NSIP non-specific interstitial pneumonia, OP organizing pneumonia.

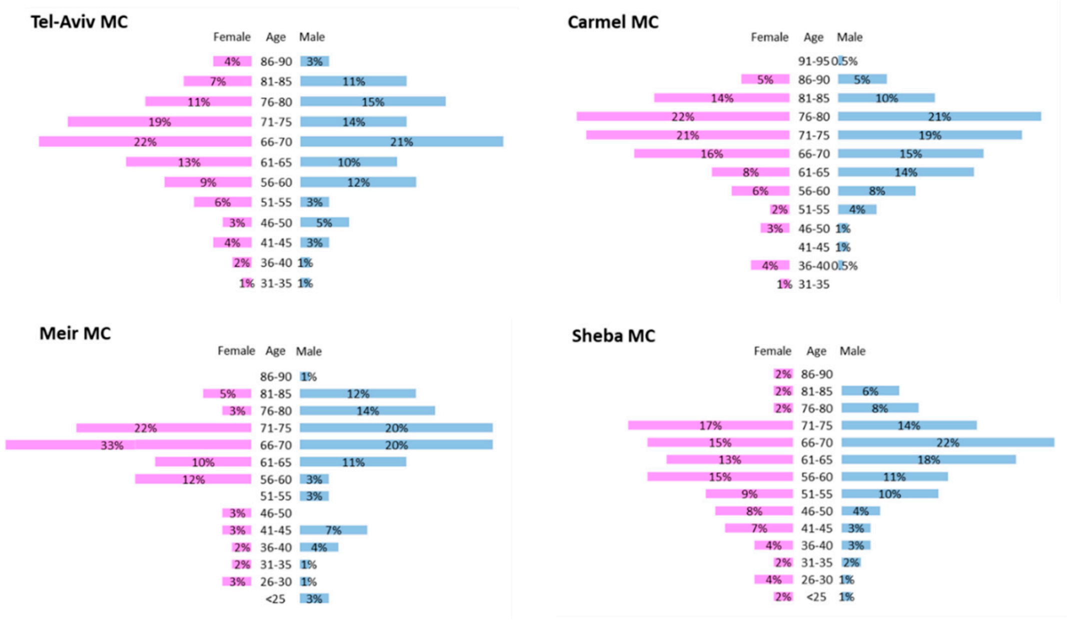

Figure S2. Age distribution for males and females in each medical center. Percentages represent the proportion of subjects belonging to each of the age subgroups.

Abbreviations: MC, medical center.

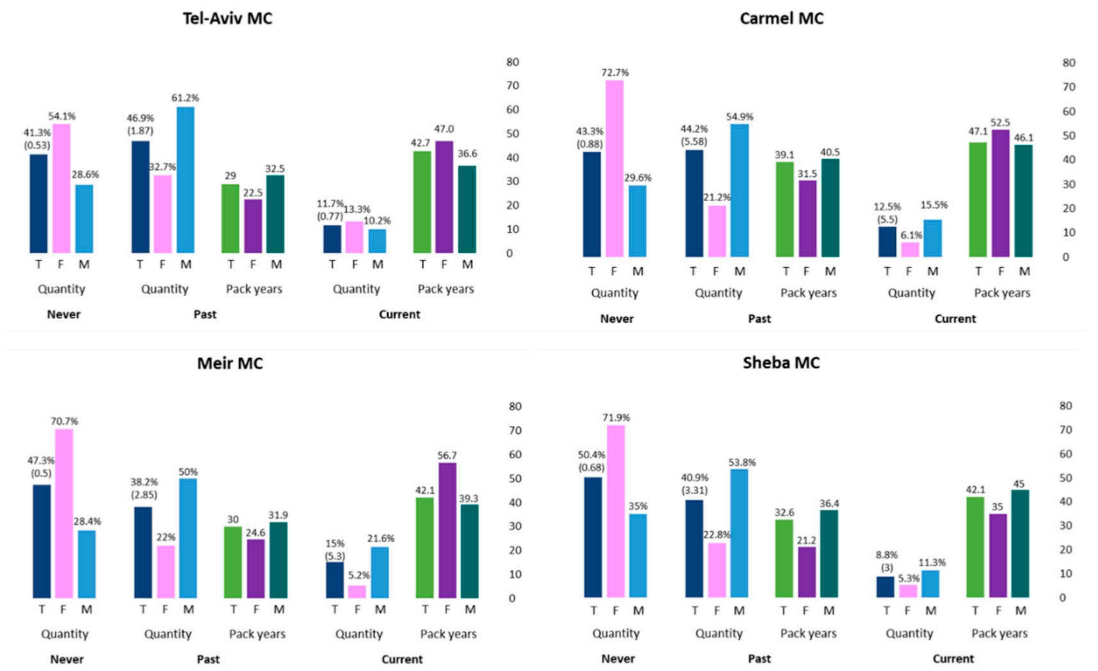

Figure S3. Smoking status and quantity per sex in each medical center. Percentages represent the proportion of subjects belonging to each of the three smoking categories (never, past, current), whereas T column is the total, F is females and M is males. Numbers in parentheses represent the male to female ratio for each smoking category. Mean number of pack years are given next to the past and current smoking categories.

Abbreviations: MC, medical center.

Table S1. Baseline characteristics of patients living in central and peripheral parts of Israel.

Variable	**Central** N=399 (%)	**Periphery** N=396 (%)	**p**
Female sex	188 (47)	129 (33)	<0.01
Age, mean (SD)#	65 (13)	69 (11)	<0.01
Ever smoker	219 (55)	221 (56)	0.79
Pack years, median (IQR)	29 (10-50)	35 (10-50)	0.01
Non-ILD lung disease	79 (20)	35 (9)	<0.01
Hypertension	150 (38)	175 (44)	0.06
Diabetes	99 (25)	102 (26)	0.76
Dyslipidemia	154 (39)	177 (45)	0.08
CVD	100 (25)	130 (33)	0.02
Heart failure	51 (13)	55 (14)	0.65
History of Cancer	76 (19)	61 (15)	0.17
**Diagnosis**			
IPF	95 (24)	210 (53)	<0.01
Hypersensitivity pneumonitis	54 (14)	9 (2)	<0.01
Pneumoconiosis	11 (3)	10 (3)	0.839
Exposure-related ILD^	65 (16)	19 (5)	<0.01
Autoimmune-related ILD	99 (25)	62 (16)	<0.01
**Pulmonary functions, mean (SD)#**			
FVC (% pred.), mean (SD) ¶	77 (22)	80 (22)	0.031
DLCO (% pred.), mean (SD) ¶	53 (21)	60 (20)	<0.01

Abbreviations: CVD cardiovascular diseases, CTD, connective-tissue disease; DLCO diffusion capacity for carbon monoxide, FVC forced vital capacity, ILD interstitial lung disease, IPF idiopathic pulmonary fibrosis, IQR interquartile range, SD standard deviation.

# Age and pulmonary functions were obtained at presentation.

¶ Missing data for FVC – 76 subjects, for DLCO – 185 subjects.

^ Including hypersensitivity pneumonitis and cases of pneumoconiosis (asbestosis, silicosis, etc.).
